# Raising awareness of antimicrobial resistance: comment on ‘Reducing expectations for antibiotics in primary care: a randomised experiment to test the response to fear based messages about antimicrobial resistance’

**DOI:** 10.1186/s12916-020-01576-z

**Published:** 2020-04-24

**Authors:** Yoshiaki Gu

**Affiliations:** AMR Clinical Reference Center, National Center for Global Health and Medicine Hospital, Tokyo, Japan

## Background

Antimicrobial resistance (AMR) is one of the greatest healthcare threats worldwide, with the number of deaths globally attributable to AMR estimated to reach 10 million per year by 2050 unless action is taken [1]. The World Health Organization published a global action plan on AMR in 2015 and called on member states to create their own action plan to tackle AMR in each country or region [2]. The first objective of the global action plan is to ‘improve awareness and understanding of antimicrobial resistance through effective communication, education and training’. A massive global public awareness campaign is thus needed to ensure proper use of antibiotics.

## Exploring effective awareness campaign

The Japanese government published a National Action Plan on AMR in 2016 that also focuses on enhancing awareness among the general public. In April 2017, the AMR Clinical Reference Center (AMRCRC) was established in Tokyo to implement the National Action Plan. One of the missions of the AMRCRC is to enhance AMR awareness among both medical personnel and the general public. The AMRCRC has conducted various campaigns via websites, social networking services, media exposure, and public events.

People’s knowledge about antibiotics and infectious diseases is not always accurate. According to a 2017 internet survey in Japan [3], nearly half of the participants answered that antibiotics kill viruses (46.8%) or are effective against the common cold and flu (40.6%). Such misconceptions can trigger inappropriate antibiotic use; some people may consult physicians just to request antibiotics (available on prescription only in Japan) for the common cold or may keep leftover antibiotics for later use themselves or to share with family members. According to a recent survey of doctors in clinics in Japan [4], half of the participants (50.4%) answered that they would prescribe antibiotics for the common cold if, regardless of explanation, patients were not convinced of the ineffectiveness of antibiotics for the condition. This highlights the importance of both AMR awareness campaigns for the general public and educational programmes for doctors.

Tsuzuki et al. reported on the factors associated with sufficient knowledge of antibiotics and AMR [5]. The use of primary information provided by healthcare professionals, research institutes, and governmental organisations was strongly associated with better knowledge of AMR and with behavioural changes regardless of educational level. Public health authorities should promote the spread of key messages on AMR and appropriate antibiotic use through their own channels and healthcare professionals.

Constructing an attractive message is crucial for public health authorities to ensure successful AMR awareness campaigns. However, it is difficult to evaluate such messages because national campaigns are conducted simultaneously using various measures. Roope et al. reported some interesting findings about the kinds of messages that are effective for enhancing public awareness [6]. They conducted internet surveys in the UK using a randomised design to compare three different messages, namely fear-only, strong-fear-plus-empowerment, and mild-fear-plus-empowerment. They found that a message based on strong-fear-plus-empowerment is likely to be more effective in decreasing the numbers of consultations and requests for antibiotics. This finding is consistent with research in other healthcare areas. Fear messages raise awareness on the issue; however, such messages might backfire if individuals are not confident enough to take the recommended actions. The combination of fear and empowerment in messages is needed for campaigns aimed at combating resistant bacteria and promoting appropriate antibiotic use.

## Conclusion

Roope et al.’s research was conducted in the UK where general practitioners work as gatekeepers in the medical system, but the results are informative for other countries, especially those where antibiotics are prescription drugs, to raise public awareness of AMR. For more effective campaigns to combat AMR, we should continue to share the experiences of different countries in tackling the problem.

## Data Availability

Not applicable

## Abbreviations

AMR: Antimicrobial resistance
AMRCRC: AMR Clinical Reference Center
